# Identification and prioritization of risks for new entrants in automobile sector using Monte Carlo based approach

**DOI:** 10.1038/s41598-024-62803-8

**Published:** 2024-05-31

**Authors:** Sarmad Farooq, Afshan Naseem, Yasir Ahmad, Muhammad Awais Akbar, Mehran Ullah

**Affiliations:** 1 Department of Engineering Management, College of Electrical and Mechanical Engineering (CEME), National University of Sciences & Technology (NUST), Islamabad, Pakistan; 2 School of Business and Creative Industries, University of the West of Scotland, Paisley, PA1 2BE Scotland, UK

**Keywords:** Risks identification, Risk prioritization, Categorization, New entrants, Automobile sector, MCS, Engineering, Mechanical engineering, Mathematics and computing

## Abstract

The automotive industry serves as a crucial support system for the economies of industrialized nations in their pursuit of international market competitiveness. Despite this industry's importance, most developing countries face the challenge of acquiring a reasonable economic position at the global level in the automotive sector for various reasons. The most salient reasons include inconsistent government policies, multiple taxes, investor insecurity, political instability, and currency devaluation. Identifying risks is crucial for a new entrant in the already-established automotive industry. The researchers have used multiple (qualitative and quantitative) techniques to identify and prioritize risks in setting up manufacturing plants. The efforts to tackle these identified risks are undertaken at the domestic and government levels to smoothen the establishment of industry. The risks are first identified, in the current study, by reviewing the previous literature and conducting interviews of the various stakeholders (automotive dealers, managers, and customers). Then this study uses Monte Carlo simulation (MCS) approach and develops a risk exposure (high, medium, or low) matrix for the automotive industry of Pakistan. The findings reveal that the depreciation of local currency against the foreign exchange, oligopoly nature of competition, and low market acceptability of new entrants due to their products' image are the most critical risks the automobile industry faces. These findings will help automotive research institutes in developing national policies that specifically aim to support new players in the automotive industry, particularly in addressing high-priority hazards. The results may also provide valuable insights for new participants seeking to identify and address the key challenges in the Pakistani automotive industry before entering it.

## Introduction

Automotive industry is one of the most important industries that draws domestic and foreign capital to help boost the country's gross domestic production (GDP). Developing countries generally lack advanced manufacturing technologies and rely on assembling vehicles instead of manufacturing operations. This results in overall slow growth and greater dependency on imported products^1^. Simultaneously, venturing into assembling business by the new entrants in developing countries is also marred with many risks^2^. The organizations can only plan their risk mitigation strategies once all the risks are recognized and categorized according to their probability and impact^3^. In a developing country with emphasis on automobile assembly operations, risks are different than the countries with manufacturing operations intensive automotive industry. The companies generally utilize outdated technology, thus lacking precision equipment's production capacity, resulting in a long production time and cost. The automotive industry of Pakistan has seen highs and lows over the past couple of decades but overall, the sector has suffered due to poor policies and negligence by decision makers^1,4^. Pakistan's automotive sector has two/three wheelers, passenger vehicles (including automobiles, jeeps, and station wagons), commercial vehicles (trucks and buses), and tractors. Being the fifth largest population in the world, the country is the hot spot for new domestic and foreign automotive companies^5^. Currently, over 25 Pakistani companies collaborate technologically with Japanese and Korean manufacturers such as Suzuki, Honda, Toyota, Hino, Hyundai, and Mazda with overall 95% market share and domination by the Three-big Japanese companies in Pakistani automotive sector (as shown in Table 1). The sector relies heavily on the imports for assembly operations. The strong presence of Japanese companies has managed to curtail the share of new entrants in the country.

**Table 1:** Existing Three-big automobile manufacturers with 95% market share [Retrieved from Sales & Production data (Jun 2021–May 2022) of vehicles published by Pakistan Automobile Manufacturers Association (PAMA)].

S. no	Name	Launched in Pak (year)	Principal JV in Pak	Plant location	Dealerships (3S) in Pak	Types of vehicles produced	Market share
1	Pak Suzuki Motor Company Ltd	1982	Pakistan Automobile Corporation	Karachi	77	Pickup/SUV/MPV/Hatchback	54%
2	Toyota–Indus Motor Company Ltd	1989	House of Habib	Karachi	46	LTV/Commercial/SUV/MPV/Sedan	27%
3	Honda Atlas Cars Pakistan Ltd	1992	Atlas Group of Companies	Lahore	35	SUV/MPV/Sedan	14%

The local companies in collaboration with Chinese automotive companies have made considerable efforts to enter in the market because of their low cost offering suitable for the local customers^6^. Therefore, these new entrants need to gauge risks for making strategies for the successful assembly and launch of their products. The identification of risks for the new entrants (other than Three-big automobile manufacturers) with 5% market share in Pakistani automotive sector (as shown in Table 2 such as joint ventures, merger, and acquisitions or venture capitalists) will certainly help all stakeholders willing to invest in automotive industry after implementation of Pakistan Automobile Development Policy (ADP) 2016–2021^7^.

**Table 2:** Details of non-Japanese (5% market share) ventures in automobile sector of Pakistan (retrieved from Sales & Production data (Jun 2021–May 2022) of vehicles published by PAMA).

S. no.	Name	Launched in Pak (year)	Principal JV in Pak	Plant location	Dealerships (3S) in Pak	Types of vehicles produced
1	Isuzu	1963	Ghandhara Industries Ltd	Karachi	36	Pickup/Truck/Bus
2	Al-Haj FAW Motors Ltd	2011	Al-Haj Group	Karachi	18	Pickup/MPV/Hatchback
3	Kia Motors Pakistan	2017	Lucky Motors Corporation	Karachi	30	SUV/MPV/Hatchback
4	Hyundai Nishat Motor Ltd	2017	Nishat Group	Faisalabad	13	LTV/SUV/MPV/Sedan
5	Prince	2017	Regal Automobile Industries Ltd	Lahore	19	Hatchback
6	DFSK	2017	Regal Automobiles Industries Ltd	Lahore	19	Pickup/SUV
7	Proton	2018	Al-Haj Group	Karachi	09	SUV/Sedan
8	Master Changan Motors Ltd	2018	Master Group of Companies	Karachi	21	Pickup/SUV/MPV/Sedan
9	United Motors	2018	United Auto Industries	Lahore	21	Hatchback
10	MG Motors	2020	JW-SEZ Group	Lahore	07	SUV
11	BAIC	2020	Sazgar Engineering Works Ltd	Lahore	32	SUV
12	Haval (GWM)	2021	Sazgar Engineering Works Ltd	Lahore	32	SUV
13	Peugeot	2022	Lucky Motors Corporation	Karachi	06	SUV

Despite concerted efforts by domestic and international automobile manufacturers, a significant majority have not successfully introduced cost-effective vehicles within the Pakistani market. A primary contributor to these shortcomings has been identified as a lack of in-depth understanding and knowledge concerning the local market dynamics and consumer preferences. A notable deficiency exists in these companies' grasp of the risks inherent to the local market context, leading to costly misjudgements. This research employs expert evaluations and the MCS technique to assess the risks confronting new entrants in the automotive sector. These risks are categorized and quantified based on exposure zones (high, medium, or low). The methodology adopted in this study offers a comprehensive risk identification framework, marking a pioneering effort within the context of the automotive industry in a developing country.

The subsequent sections of the paper detail the development of the proposed framework followed by presenting the results of an experimental evaluation for its efficacy.

## Literature review

### Global automotive industry

Throughout history, car production has significantly impacted early industrialised nations' economic growth. This industry has introduced advancements in technology and workplace structure that have improved productivity and have been adopted by other sectors. The global automobile sector is known for its intricate and comprehensive integration of global, regional, national, and local value chains, resulting in a unique pattern of global integration. The industry is continuously evolving, as evidenced by car-to-external object communication technology developments, car sharing, and the transition to electric automobiles and autonomous driving. The industry is crucial in driving global economic growth, fostering technical advancements, and influencing employment rates. Germany has remained the leading vehicle exporter for almost a decade, while China holds the top in automobile production. The United States, Japan, and Germany rank below China in car production. The sector's trajectory is influenced by the growing manufacturing of electric and hybrid vehicles, progress in self-driving technology, the growth of car sharing services, and rapid breakthroughs in automobile-related software^8,9^. As a result of the industry's economic multiplier effects and high labor productivity, contemporary emerging economies, including India and Vietnam view the establishment of an automotive sector as a crucial industrialization milestone^10^. Encouraging the usage of new energy vehicles (NEVs) in big cities is not only a key area of priority for the government to encourage the transformation of the automobile industry, but it is also a crucial step in reducing environmental pollution^11^, unlike Pakistan where people still prefer conventional/combustion vehicles due to many reasons^12^. The state typically has a leading role in promoting efforts to establish the industry in new countries^13^.

### Risk identification tools and techniques

Risk identification is the first step in the risk assessment process^14,15^. Automobile manufacturing industry needs to identify the risks so appropriate strategies may be developed for tackling them^16^. Risk assessment techniques include both qualitative and quantitative methods (Table 3) to identify and describe the risks associated with a decision problem and analyse the potential impacts of the risk^17^.

**Table 3:** Risk identification tools and techniques^17^.

Project-specific documents	Programmatic documents	Techniques
Project description	Historic data	Brainstorming
Work breakdown structure	Final project reports	Scenario planning
Cost estimate	Risk response plans	Expert interviews
Design & construction schedule	Organized lessons learned	Nominal group methods
Procurement plan	Published commercial databases	Delphi technique
Listing of team's issues and concerns	Academic studies	Influence or risk diagramming

### Risk categorizing techniques prevalent in the academia and industry

Generally, processes of qualitative and quantitative risk analysis can be used independently or together as shown in Table 4. Deliberations of time and budget disposal and the need for qualitative or quantitative reports about risk and impacts determine which methods are better to use^17^.

**Table 4:** Generic risk analysis tools and techniques^17^.

Qualitative	Quantitative
Risk probability and impact assessment	Data gathering and representation technique
Probability and impact matrix	Quantitative risk analysis and modeling techniques
Risk data quality assessment	Sensitivity analysis
Risk categorization	Expected monetary value (EMV) analysis
Risk urgency assessment	Decision tree analysis
Expert judgement	Expert judgement

A. del Cano and M. Pillar de la Cruz (2002) also divided risk analysis techniques into qualitative and quantitative^18^ as shown in Table 5.

**Table 5:** Qualitative and quantitative techniques used in risk analysis.

S. No	Sectors	Qualitative	Quantitative	Country	References
1	Construction	Evaluation of qualitative risk analysis technique through questionnaires/interviews	–	Nigeria	Adedokun et al.^19^
2	Renewable energy technology	–	Fuzzy analytical hierarchy process (FAHP)—Technique for Order Preference by Similarity to Ideal Solutions (TOPSIS)	Iran	Sadat et al.^20^
3	Sugar industry	–	AHP—Best worst method (BWM)	India	Bathrinath et al.^21^
4	Construction (tunnel boring)	–	Event Tree Analysis—Fault Tree Analysis	Korea	Hong et al.^22^
5	Construction	–	Fuzzy AHP—Project Risk Analysis Tool (PRAT)	Greece	Koulinas et al.^23^
6	Textile industry	–	AHP—TOPSIS	India	Bathrinath et al.^24^
7	Leather industry supply chain	–	BWM—Pareto analysis	India	Moktadir et al.^25^
8	Sanitation system	Fuzzy set qualitative comparative analysis	–	USA	Davis et al.^26^
9	Autonomous vehicles	–	AHP—TOPSIS—Multi-Criteria Optimization and Compromise Solution (VIKOR)	Turkey	Bakioglu and Atahan^27^

Comparison of risk analysis tools and techniques with their properties such as their adaptation to risk matrices, assessment of risk exposure, probabilistic relative importance of risks, prioritization of risks and categorization of risks into multiple exposure zones is tabulated in Table 6.

**Table 6:** Comparison of risk analysis techniques.

Technique	Risk matrices adaptation	Risk exposure assessment	Risks’ probabilistic relative importance	Risks’ prioritization	Risks’ categorization into multiple exposure zones	References
Interpretive structural modelling	✗	✗	✗	✗	✗	Abbasi and Ryan^28^
Fuzzy AHP	✗	✗	✗	✗	✗	Ribas et al.^29^
Statistical analysis	✓	✗	✗	✗	✗	Wu et al.^30^
AHP	✓	✗	✗	✗	✗	Macharis et al.^31^
Social network analysis	✗	✗	✗	✗	✗	Yuan et al.^32^
MCS	✓	✓	✓	✓	✓	This study

### Risk simulation techniques and why Monte Carlo is best for such studies?

It is a stochastic simulation method consists of the stages in sequence as: (i) identification of criterion and relevant variables, (ii) allocation of probability for relevant variables, (iii) determination of correlation coefficient among relevant variables, (iv) and simulation execution and results analysis. The MCS method is a stochastic simulation method used in the quantitative risk analysis. It has a wide range of application in various fields: numerical mathematics, physical chemistry, statistical physics, computer graphics, finance etc^33^. Strengths, weaknesses, opportunities, and threats (SWOT) analysis of MCS in comparison with some other multi criteria decision making (MCDM) tools and techniques are tabulated in Table 7.

**Table 7:** SWOT analysis of MCS with few MCDM tools and techniques for risk prioritization.

MCDM tools/techniques	Strengths	Weaknesses	Opportunities	Threats
AHP	Often combined with other methods Faster comparing with other MCDM methods Method has comprehensible logic Widely applied for technology evaluation and place selection^34^.	Further analysis is needed to verify the results^34^.	Advisable to combine with sensitivity analysis^34^.	Different hierarchies of criteria may influence the difference in allocation of weights^34^.
Fuzzy sets	Provides a solution to handling subjective Uncertain data There is no limit of criteria Makes the comprehensiveness of the decision-making process stronger Often combined with other methods^34^.	An additional measure, requires further calculations^34^.	Possible to combine more with other methods, not only TOPSIS and AHP^34^.	Not observed
TOPSIS	Concept depicted in a simple mathematical logic Computation process is straightforward Easy application Consistency and reliability Faster Often combined with other methods^34^.	But it only considers the closeness between each alternatives, and does not taking the decision makers’ risk aversion attitude into account^35^.	Method can be easily adapted to solve sustainability issues^34^.	Not observed
VIKOR	Adapted to solve quite different problems Tolerant to deviations of values in the assessment period Popular to combine with other methods^34^.	Narrow experience in the field of energy^34^.	Useful to apply and compare the results from different methods to achieve strength reliability of the assessment^34^.	Possible errors in calculations^34^.
Full Multiplicative Form of Multi-Objective Optimization by Ratio analysis (MULTIMOORA)	Tolerant to deviations of values in the assessment period Consistency and reliability Consists of mathematical based techniques^34^.	Quite long computation process comparing with other MCDM methods^34^.	Easily adaptable to solve different sustainability issues^34^.	Possible errors in calculations due to sophisticated computation process^34^.
MCS	Assesing uncertainty in complex distributions of input variables, including asymmetric and U-shaped distributions among others. The need to calculate partial derivatives has been eliminated, simplifying the process of evaluating uncertainty. Evaluating the effective degrees of freedom is not necessary. The coverage interval is derived using an alternative method that specifies the coverage probability. The assumption regarding the distribution of measurand is not required. It is applicable to models that are ‘‘black boxes’’ where the equations are not in the form y = f(x_1_, …, x_n_). These ‘‘black boxes’’ can be highly complex and include varying levels of detail. If the model can be evaluated, then uncertainty can be simulated^36^.	It necessitates software that is compatible and capable of generating the necessary random numbers (2E5) in a reproducible and statistically robust manner. A computer with adequate speed is essential, which might not be accessible to all the researchers^36^.	It relies on the dissemination of distributions, which sets it apart from other approaches that depend on dissemination of uncertainties. Ideal for scenarios where the model represents significant nonlinearity in characteristics. Calculating the partial derivatives and effective degrees of freedom is not required. Processing of interdependent input variables and modelling of an infinite number of output quantities is possible^36^.	An incorrect choice of Probability Density Function for the input parameters could lead to ambiguous results. In some cases, where the model equations do not have the parameters such as the impact of environmental conditions, uncertainty due to resolution, bias, and so on. It can be executed multiple times to verify the reproducibility of the findings. The choice of trials [2E5] is often subjective and usually depends on the operator. Ideally, it should be application dependent^36^.

There is a study by Garg et al., in which microphone free-field calibrations in the frequency range from 125 Hz to 20 kHz were investigated by utilizing a comparison of Guide to Expression of Uncertainty in Measurement and MCS approaches. They used an Excel spreadsheet to implement this and used a mixture of Gaussian and rectangular probability density functions to model uncertainty inputs. The uncertainty results obtained by both these methods were very close at two frequencies when higher-order nonlinear and correlation effects were absent. Rachakonda et al. concluded that the MCS approach may be preferred to get a convenient method for calibration measurement capability assessments for calibration and industrial measurement laboratories^37,38^.

MCS operationalizes a new process for prioritizing sustainability-related project risks using risk matrix data. It retains conflicting assessments of experts about the probability and impact ratings of sustainability-related risks and establishes the ranking of risks relative to the decision-maker's risk appetite. The main merit of the proposed process is its compatibility with the conventional risk matrix-based approaches since risk matrix data are used to operationalize the process and the decision-maker's risk appetite is established across risk exposure zones within a risk matrix. The compatibility of new risk assessment techniques with the existing practices is essential since "… it seems that they (practitioners) prefer to use the simple quantitative tools to perform a quantitative risk analysis. Such a conclusion might need to be always considered when introducing any alternative to the existing tools"^39^.

The risks in automotive sector of Pakistan highlighted in this study are based on literature review as well as semi-structured interviews by the main stake holders of the industry. So, it is good blend of both previous work and current situation in Pakistan to assess the risks for new entrants in the automobile sector. MCS provides the solution to prioritize those risks and uncertainties to make better decision making by the new entrants in such a market of a developing country. According to Akbar et al., MCS is utilized due to its speedy calculations and probability distributions (iterations in thousands) to get the results^40^ in such a scenario.

### Case of Pakistan automotive industry

In Pakistan, vehicle production data (from 1974 to 75 to the present) shows that the industry performed its best in the 1970s and then again in the 2000s. Performance plunged in the 1980s and fell even worse in the 1990s, to less than 4%^41^. Pakistan's automobile sector has always failed to fulfil its full potential except for the motorcycle manufacturing industry. Most automobile assemblers use technology and provide minimal features compared to cars on the global market. Domestic consumers cannot access the most advanced technology and automobile safety standards. In developing countries like Pakistan, the automobile sector is facing challenges in growing under its stressed economy. For the survival and advancement of vehicle sector, the current era of globalisation necessitates fashionable models, enhanced fuel efficiency, cost reduction, and enhancement in consumer comfort. This competitive period necessitates more attention to safety and the environment. For any developing countries, auto industry is one of its emerging industries. Vehicle manufacturers globally putting significant efforts to spend their capital on the development and automation of assembly plants to find smart mechanism bringing value to their investment and contribution in the sector. According to the ADP 2016–2021 by Economic Coordination Committee (ECC) of Pakistan, a new manufacturer requires special treatment and better incentives to promote and initiate its product, create its reputation, form a network for expanded services, and establish a base for part manufacturers.

This rationalizes the formulation of the following two important research questions answered through this research.

RQ1: What risks are associated with the new entrants in the Pakistani automobile Industry?

RQ2: What are the significant risks (that lay in high exposure zone) for the new entrants in the automobile sector of Pakistan?

## Research methodology

A mixed research approach is used to answer the research questions. In this research, interviews were conducted using a qualitative method while a quantitative questionnaire (Annexure-'A'), using a 5-point Likert scale, was used to acquire the probability and impact factor ratings for the identified risks. These ratings were then used to calculate the total risk exposure of the individual risk.

Thirteen experts/participants were approached to identify the potential risks through semi-segmented interviews (Table 8). After the initial interviews and identification of risks, 30 experts from the relevant sector were approached to respond to the qualitative questionnaire (Annexure-'A'), thus determining the probability and impact of risk factors. Chief Executive Officers (CEOs)/heads of departments (HoDs), purchasing managers, and operational managers from the company authorized 2S/3S dealerships of the automotive industry comprised the experts.

**Table 8:** Demographics of interviewees.

Interviewee(s)	Designation	Industrial experience (years)
Interviewee 1	Authorized 3S Dealership Manager	8
Interviewee 2	Head of Sales & Marketing	11
Interviewee 3	Dealership Owner	16
Interviewee 4	Dealership Partner & Manager	12
Interviewee 5	HoD Sales	17
Interviewee 6	Assistant Manager Sales & Marketing	4
Interviewee 7	Managing Director	10
Interviewee 8	Assistant Manager Sales & Marketing	7
Interviewee 9	Assistant Manager Sales & Marketing	2
Interviewee 10	CEO	1
Interviewee 11	Assistant Manager Sales & Marketing	16
Interviewee 12	Senior Manager Sales & Marketing	17
Interviewee 13	Manager Sales	1

The interview technique enabled for the deeper insight provided by the participants in the relevant industry to be captured, and the findings in a study go beyond the need to gather quantitative data. Semi-structured interview approach was chosen at this stage based on the research objectives and a literature evaluation. The methodology was compared to similar earlier studies by considering their respective research techniques and tools. Similarly, a list of conducted interviews and survey questionnaire distribution details was summarized to aid in identifying risks for new entrants in the automobile sector. The interview questions were open-ended and not only limited to research-related information.

There are 29 risks (in total) out of which 12 are identified through a literature review (Table 9) whereas 17 are identified through semi-structured interviews conducted in the automotive industry of Pakistan (Table 10).

**Table 9:** Identification of risks through literature review.

S. no	Risk ID	Identified risks
1	R1	Inconsistent government policies^42,43^
2	R2	Rupee devaluation against foreign currencies^44,45^
3	R3	High/multiple taxation^5,44,46^
4	R4	High production cost^47^ of locally assembled vehicles compared to other markets^45^
5	R5	No local manufacturing^42,44,45,48^
6	R6	Less number of vendors^42^
7	R7	Supply and demand issues^44^
8	R8	Oligopoly^44,49^
9	R9	Lack of skilled manpower^42,44^
10	R10	Brand-loyal automotive market^48^
11	R11	Price sensitive market^42^
12	R12	COVID-19^44,45^

**Table 10:** Identification of risks through semi-structured interviews.

S. no	Risk ID	Identified risks	Interviewee(s) no. (see Table 8 for details)
1	R13	Political instability	3, 4, 5, 9, 10, 11, 13
2	R14	Political influence	1, 3, 4, 5, 6, 7, 8, 9, 10, 11, 12, 13
3	R15	No finance and stock audit	11, 13
4	R16	No export policy	11, 12
5	R17	Non-compliance of standards	11
6	R18	Investor insecurity	3, 5, 6, 9
7	R19	No infrastructure for electric vehicles	2, 6, 7, 9
8	R20	Competition	5, 7, 8, 9, 10, 11, 13
9	R21	High cost of raw materials	4, 5, 9, 13
10	R22	Bribery	1, 2, 3, 4, 5, 6, 10, 11
11	R23	Principal JV repute	1, 4, 5, 6, 7, 8, 10, 12, 13
12	R24	Own money	1, 3, 4, 9, 11, 12, 13
13	R25	Shortage of electronic chips used in engine control units (ECUs)	4, 13
14	R26	Compromised spare parts availability	1, 3, 7
15	R27	SUV market overrun	11
16	R28	Bad image of Chinese products	1, 2, 3, 4, 7, 8, 12, 13
17	R29	Low purchasing power of end consumer	4, 7

This research used a mixed-method approach, a blend of qualitative and quantitative methodologies as per Morgan's (1998) priority sequence model^50,51^. Firstly, researchers began with a qualitative study by conducting semi-structured interviews to identify risks based on interviewees' industrial experience. Then, a quantitative questionnaire (Annexure-'A') with 29 items (risks) based on a 5-point Likert scale was distributed in the relevant industry, asking to score probability and impact for each risk. This method was used to decide based on the discrete values of each risk, which enables precise dynamic simulations^52^.

Risk exposure is found out by multiplying the risk probability and risk impact rating for individual risks as shown in Eq. (1) ^3^ below:

1 $${\text{R}}_{{{\text{Ei}}}} = {\text{ P}}_{{{\text{Ri}}}} \times {\text{ I}}_{{{\text{Ri}}}}$$

where R_Ei_ is the risk exposure, P_Ri_ is the probability of risk, and I_Ri_ is the impact of risk.

for i = 1, 2, 3, …, n

During MCS, the probabilities and impact factor ratings of the identified risks were simulated and modelled by using @Risk version 8.2 (a Microsoft Excel add-in by Palisade).

## Data analysis

A total of twenty-nine risks (R1, R2, …, R29) were identified in the study from the literature review/published interviews worldwide and by conducting the semi-structured interviews, and their risk exposure and risk signature values are shown in Table 11. The risks were classified into three risk exposure zones: i.e., Low risk exposure, medium risk exposure, and high-risk exposure. The risks which have an exposure value below 16 lay in low-risk exposure zones, between 16 and 20 lay in medium risk exposure zone, and between 20 and 25 lay in high-risk exposure zone.

**Table 11:** Risk exposure and risk signature values.

Risk ID	R_Ei_ (before MCS)	R_Ei_ (after MCS)	Risk signature values (probability to lay in high-risk exposure zone)	Risk signature values (probability to lay in medium risk exposure zone)
R1	14.387	12	20.2	19.4
R2	19.938	25	70	10.9
R3	19.060	20	62.4	27.5
R4	10.181	16	5.9	14.8
R5	18.370	16	52.5	25.8
R6	9.185	20	4.3	8.7
R7	14.958	12	25.1	18.7
R8	11.027	25	7	17.2
R9	11.428	16	8.6	17.2
R10	13.168	8	23	17
R11	13.666	15	17.9	24.6
R12	10.933	10	9.2	9.1
R13	13.438	16	23.5	10.1
R14	12.369	16	9.8	18.5
R15	16.416	16	27.9	43.4
R16	8.418	16	0	5.9
R17	11.325	4	11.1	9.5
R18	16.129	12	27.8	30.8
R19	4.1330	2	0	0
R20	12.089	6	10.5	17
R21	10.990	16	3.5	19.2
R22	12.665	6	12.6	19.4
R23	9.557	12	0	13.3
R24	12.949	15	17.5	13.7
R25	6.051	16	0	1.6
R26	9.506	6	0	5.8
R27	5.2860	4	0	0
R28	10.956	25	6.6	10
R29	6.493	9	0	1

### Conventional risk prioritization

The conventional risk prioritization matrix is a system in which the risk exposure is calculated by multiplying the individual risk priority and impact rating, and the risk exposure values determine the severity of the individual risk factors. The graph can be plotted against the risk exposure values and severity of individual risks can be identified, and the mean value is calculated from the given data set to measure the total risk exposure. This system of measuring risks has certain limitations as it can only rely on the data collected without any simulations and simply plots the graph of the mean values of risk exposure of individual risks.

**Figure 1:**
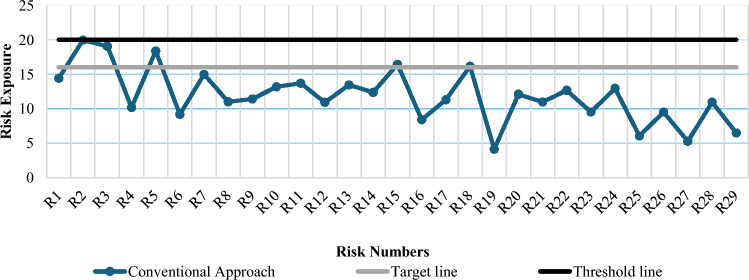
Graph of risk exposures using mean values (conventional approach).

From data of this study, when plotted against the mean values using the conventional risk prioritization approach, the data shows that only one risk R2 (Rupee devaluation against foreign currencies) touches the threshold of high-risk exposure zone, and no other risk lay in high-risk exposure zone. Four risks lay in medium risk exposure zone and twenty-four in low-risk exposure zone, as shown in Fig. 1. From the graph, it can be concluded that this conventional method for risk prioritization is not enough to accurately and precisely measuring the exact risk exposure value. Thus, another technique should be applied to measure the risk exposure value that is accurate and precise.

### MCS based risk exposure prioritization

As discussed above, the conventional approach to accurately measure the risk exposure value is not well observed and does not provide a clear picture of the exact risk exposure. As discussed earlier, the MCS has been used by using @Risk version 8.2 (a Microsoft Excel add-in by Palisade). The data were then transferred into an excel sheet and MCS were run on the probability ratings, impact ratings and the total risk exposures of individual risks. There were 1300 simulations after that the convergence have been achieved.

**Figure 2:**
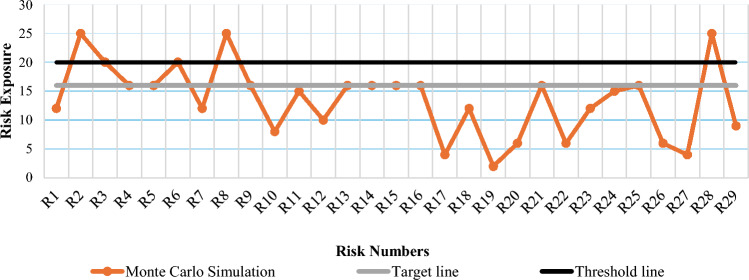
Graph of risk exposures based on MCS.

After the MCS, the results show that five risks lay in high-risk exposure zone, nine in medium risk exposure zone and the rest in low-risk exposure zone as shown in Fig. 2. These results show a significant difference in the conventional risk exposure matrix using means and MCS based risk exposure prioritization matrix.

The risk exposure profiles were used to identify which risks lay in high, medium, and low exposure zones as shown in Table 12. Five risks such as R2 (Rupee devaluation against foreign currencies), R3 (High/Multiple Taxation), R6 (Low vendor availability), R8 (Oligopoly), and R28 (Bad image of Chinese products) lays in high-risk exposure zones, which means these risks are the most critical risks faced by the automobile industry of Pakistan (especially new entrants).

**Table 12:** Risks in high, medium and low risk exposure zones.

Risk exposure Zones	Risk exposure value	Risks' ID	Frequency
High risk Exposure zone	20–25	R2, R3, R6, R8, R28	05
Medium risk exposure zone	16–20	R4, R5, R9, R13, R14, R15, R16, R21, R25	09
Low risk exposure zone	< 16	R1, R7, R10, R11, R12, R17, R18, R19, R20, R22, R23, R24, R26, R27, R29	15

## Results and discussion

Based upon the responses by the subject matter experts, mean and standard deviation values along with their probability distribution of probability rating as well as impact rating for every identified risk (R1, R2, …, R29) is shown in Table 13. According to Barreras, the most common number for simulation repetitions is 1000 iterations^33,53^ likewise authors did with MCS for all risks (R1, R2, …, R29), calculated by @Risk version 8.2 (a Microsoft Excel add-in by Palisade), is considered the true values for further assessment of the risks' prioritization.

**Table 13:** Statistics and distributions for automobile sector risks (idea is conceived from Qazi et al.^39^).

Risk ID	Probability rating			Impact rating			Risk profile (MCS)
	Mean	Standard deviation	Probability distribution	Mean	Standard deviation	Probability distribution	
R1	3.56	0.883		4.03	0.65		
R2	4.46	0.67		4.46	0.67		
R3	4.26	0.62		4.46	0.49		
R4	3.30	1.10		3.06	0.96		
R5	4.19	0.71		4.37	0.54		
R6	2.86	1.08		3.23	1.08		
R7	3.80	0.70		3.93	0.77		
R8	3.40	0.95		3.23	0.91		
R9	3.26	1.09		3.50	0.80		
R10	3.63	1.08		3.63	1.07		
R11	3.56	0.99		3.83	0.63		
R12	3.29	1.03		3.29	1.03		
R13	3.66	0.97		3.66	0.97		
R14	3.50	0.92		3.53	0.71		
R15	4.06	0.63		4.03	0.54		
R16	2.96	0.75		2.83	0.77		
R17	3.36	1.13		3.36	0.94		
R18	4.10	0.65		3.93	0.67		
R19	1.90	0.70		2.16	0.68		
R20	3.50	1.02		3.46	0.76		
R21	3.33	0.82		3.30	0.93		
R22	3.36	0.98		3.76	0.80		
R23	2.86	0.95		3.33	0.59		
R24	3.56	0.92		3.63	0.87		
R25	2.30	0.93		2.63	0.79		
R26	3.06	0.62		3.10	0.53		
R27	2.29	0.58		2.30	0.45		
R28	3.40	0.88		3.23	0.84		
R29	2.29	0.78		2.83	0.82		

It can be observed from reviewing the results from the conventional risk prioritization approach and using MCS based risk prioritization approach that both have significant difference in results. It is observed that by using conventional approach and plotting the graph against mean values of risk exposure, that only one risk R9 (Rupee devaluation against foreign currencies) lays in high-risk exposure zone and no other risk lays in high-risk exposure zone, whereas the results from the MCS shows that five risks lay in high exposure zone.

**Figure 3:**
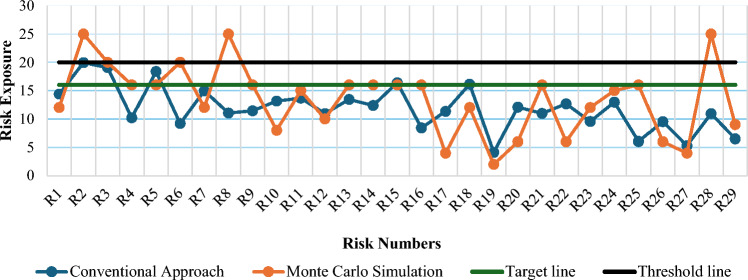
Comparison between conventional approach and MCS results in automotive industry of Pakistan.

Five risks i.e., R2 (Rupee devaluation against foreign currencies), R3 (High/Multiple Taxation), R6 (Low vendor availability), R8 (Oligopoly), and R28 (Bad image of Chinese products) that were not seemed to be of critical nature using conventional approach but found as critical by the MCS as evident in Fig. 3. Rupee devaluation as compared to US$ and always behaving inconsistent since few years in the country plays a vital role to attract/detract foreign investment, also multiple taxation costing raw material import by the government of Pakistan discouraged potential manufacturers to invest here. Nine risks such as R4 (High production cost of locally assembled vehicles compared to other markets), R5 (No local manufacturing), R9 (Lack of skilled manpower), R13 (Political instability), R14 (Political influence), R15 (No finance and stock audit), R16 (No export policy), R21 (High cost of raw materials), and R25 (Shortage of electronic chips used in engine control units) are found as medium risk exposure zones by MCS-based results. High utilities rates contribute high production cost in Pakistan as a challenge for investors to meet their production expectations. Due to economic situation in the country, brain-drain is damaging the presence of skilled manpower, leaving behind unskilled manpower. Political instability in Pakistan has always been a debate by foreign OEMs prior to their investment in the country.

By using the conventional approach, it is observed that 24 risks lay in low-risk exposure zone, whereas MCS-based results show that 15 risks in low-risk exposure zone.

## Conclusion

This research aims to outline and classify risks into high, medium, and low exposure categories, thereby providing a framework for guidance to new entrants within the automotive sector in Pakistan. The study employs the MCS methodology to assess and quantify the likelihood of risks and identify factors that could potentially influence the automotive industry in a developing country context. By leveraging statistical sampling techniques, this method facilitates the quantification of risk and the forecasting of possible project outcomes, enabling the determination of risk probabilities. By acquiring insights regarding potential hazards and their respective exposure levels, organizations are equipped to enhance the efficiency of resource allocation. Furthermore, the applicability of this method extends beyond the automotive industry, offering potential benefits to other sectors characterized by similar operational dynamics.

The study also undertakes a comparative analysis between the conventional risk matrix-based rating methodology and the MCS-based approach within the context of risk assessment. These methodologies exhibit significant disparities in the identification and evaluation of primary hazards. For instance, when employing the average value traditional risk rating system, certain risks such as inadequate vendor availability, elevated or multiple taxation levels, market oligopoly, and the negative perception of Chinese automobile manufacturers were not deemed critical. Conversely, the application of the proposed MCS-based method identified these risks as paramount hazards. Such differences underscore the importance of adopting a comprehensive and systematic methodology to model and analyze risk exposure in the automotive industry. Moreover, the proposed approach is broadly applicable for assessing risk exposure across various risks associated with the manufacturing sector The preemptive understanding and mitigation of potential hazards, in the context of investment within any given sector, are imperative for decision-makers aiming to navigate challenges effectively. This proactive risk management is deemed fundamental for the sustainability of a business. In order to maintain a competitive edge, organizations are required to thoroughly assess prospective risks in advance, thereby determining which risks are within their tolerance threshold and which are not. Such strategic risk assessment and decision-making processes are critical for ensuring a company's long-term viability and success in a dynamic and uncertain market environment.

In developing countries, almost every industry is facing issues for investment opportunities. The study's findings have major benefits for all automotive manufacturers and government agencies of those underdeveloped nations. It serves the automotive manufacturers through which they would be able to prioritize their investment opportunities, such as for the government policy-making agencies, in such a way that the model is useful in picking out better alternatives to ease the business practices for the automobile manufacturers by their own risk setting and preferences. The study's findings show that the most critical risks of the automobile industry that should be considered are currency devaluation, low vendor availability, high taxation, supply and demand issue and bad repute of Chinese manufacturers. The technique employed can aid researchers to understand better how various risks can impact new entrants' investment decisions. With this model, practitioners can prioritize and categorize risks.

## Managerial implications

This study presents a new framework, based on the MCS method, that provides engineering managers with an improved strategy for prioritizing risks. Conventional risk assessment methods have shown inadequacies, especially in their capacity to identify and analyze potential significant threats precisely. The suggested method addresses these issues by gathering substantial information from key individuals. This ensures that the risk prioritization matrix is created thoroughly and relies on agreement among all parties involved. This comprehensive approach enables a more precise identification of crucial hazards and improves the managerial process of evaluating and handling risks.

Crucially, for the model to be effective, it is necessary to obtain comprehensive probability and impact evaluations for each risk from experts in the field. Thorough evaluations like this help create customized plans to reduce risks, enabling firms to allocate resources effectively based on their specific risk preferences. Furthermore, due to the ever-changing nature of risk factors, it is necessary to regularly update the risk assessment to maintain the prioritising matrix's relevance.

The suggested risk identification and categorization framework is highly versatile, having the potential to be applied in a wide range of sectors and projects. Future study should investigate comparison analyses to determine the effectiveness of this model compared to traditional risk assessment methodologies. This approach adds to the wider academic discussion on risk management and offers practical and actionable insights for organizational leaders who want to negotiate the complexities of risk in a constantly changing business environment.

## Future directions

The suggested approach concentrates solely on the risk assessment step, with no consideration given to evaluating various risk mitigation measures. Some more advanced methods (like ordered weighted averaging (OWA), and Decision making trial and evaluation laboratory (DEMATEL) analysis etc.) can be adopted for this study by utilizing their strengths to compare the results and findings. Allocation of resources to risks using the normalized criticality index can be done to mitigate them. This study uses the negative connotation of risk, dismissing the upward potential of risk as an opportunity. Individual hazards are assumed to be independent elements, which appears to be a widely accepted simplification of the real-world problem of risk interdependence. Individual hazards are given discrete probability distributions rather than continuous ones. This research might be developed along several paths of investigation. The suggested approach may be expanded to include evaluating prospective risk mitigation solutions across multiple hazards. To describe positive and negative correlations across numerous hazards, both positive and negative connotations of risk might be considered. The suggested approach may be expanded to account for interdependence among individual risks affecting social, environmental, and economic aspects of the sustainability of any sector. Expert and risk matrices data can be turned into continuous distributions. Moreover, this study only incorporates passenger cars and does not account for trucks, buses, 2 / 3 wheelers, or tractors, which are also major contributing pillars of the automobile industry; future studies may incorporate these sectors while researching the automobile industry.

### Supplementary Information

Supplementary Information.

## Data Availability

The datasets used and/or analysed during the current study available from the corresponding author on reasonable request.
