# Supplementary material for: Identification and prioritization of risks for new entrants in automobile sector using Monte Carlo based approach
Source: Sci Rep. 2024 May 31;14:12571. doi: 10.1038/s41598-024-62803-8 (PMC11143293; doi:10.1038/s41598-024-62803-8)
Supplement: Supplementary file 1 — Supplementary Information. [file 41598_2024_62803_MOESM1_ESM.docx]

**Annexure- 'A'**

**Questionnaire for Risks Identification for New Entrants in Automobile Sector in Pakistan**

You are invited to participate in this research project which is being undertaken for academic purposes. The topic of my research is "**Identification and categorization of Risks for new entrants in Automobile sector of Pakistan**".

Please read carefully and answer all questions as accurately as possible. You are assured of anonymity. No information you provide will be disclosed to anyone and will be used only for research purposes. Do not write your name anywhere on the questionnaire. Please do not leave any statements blank and respond to all statements.

**Designation: _____________________ Experience: ______________________**

Please tick (√) the options given against probability and impact ratings of identified risks.

| **S. No.** | **Risk Factors** | **Probability Rating** | | | | | **Impact Rating** | | | | |
| --- | --- | --- | --- | --- | --- | --- | --- | --- | --- | --- | --- |
|  |  | **1** | **2** | **3** | **4** | **5** | **1** | **2** | **3** | **4** | **5** |
|  |  | **Very Low** | **Low** | **Normal** | **High** | **Very High** | **Very Low** | **Low** | **Normal** | **High** | **Very High** |
| 1. | No local manufacturing |  |  |  |  |  |  |  |  |  |  |
| 2. | Inconsistent government policies |  |  |  |  |  |  |  |  |  |  |
| 3. | Bad image of Chinese products |  |  |  |  |  |  |  |  |  |  |
| 4. | Price sensitive market |  |  |  |  |  |  |  |  |  |  |
| 5. | Lack of skilled manpower |  |  |  |  |  |  |  |  |  |  |
| 6. | Less number of vendors |  |  |  |  |  |  |  |  |  |  |
| 7. | Brand loyal automotive market |  |  |  |  |  |  |  |  |  |  |
| 8. | Political instability |  |  |  |  |  |  |  |  |  |  |
| 9. | Rupee devaluation |  |  |  |  |  |  |  |  |  |  |
| 10. | High taxation |  |  |  |  |  |  |  |  |  |  |
| 11. | High prices |  |  |  |  |  |  |  |  |  |  |
| 12. | COVID-19 |  |  |  |  |  |  |  |  |  |  |
| 13. | Raw material costs high |  |  |  |  |  |  |  |  |  |  |
| 14. | Supply and demand issue |  |  |  |  |  |  |  |  |  |  |
| 15. | Monopoly of already established companies |  |  |  |  |  |  |  |  |  |  |
| 16. | Bribery Culture |  |  |  |  |  |  |  |  |  |  |
| 17. | High Competition |  |  |  |  |  |  |  |  |  |  |
| 18. | Low purchasing power of customer |  |  |  |  |  |  |  |  |  |  |
| 19. | Principal JV repute |  |  |  |  |  |  |  |  |  |  |
| 20. | Own culture |  |  |  |  |  |  |  |  |  |  |
| 21. | Investor insecurity |  |  |  |  |  |  |  |  |  |  |
| 22. | Political influence |  |  |  |  |  |  |  |  |  |  |
| 23. | Electronic Chips shortage |  |  |  |  |  |  |  |  |  |  |
| 24. | Spare parts availability |  |  |  |  |  |  |  |  |  |  |
| 25. | No finance and stock audit |  |  |  |  |  |  |  |  |  |  |
| 26. | No standards compliance |  |  |  |  |  |  |  |  |  |  |
| 27. | No export policy |  |  |  |  |  |  |  |  |  |  |
| 28. | EV as a threat |  |  |  |  |  |  |  |  |  |  |
| 29. | SUV market overrun |  |  |  |  |  |  |  |  |  |  |
